# The germ theory revisited: A noncentric view on infection outcome

**DOI:** 10.1073/pnas.2319605121

**Published:** 2024-04-05

**Authors:** Fredric Carlsson, Lars Råberg

**Affiliations:** ^a^Department of Biology, Lund University, Lund 223 62, Sweden

**Keywords:** germ theory, Koch’s postulates, infection outcome, host–pathogen interaction, immunodeficiency

## Abstract

The germ theory states that pathogenic microorganisms are responsible for causing infectious diseases. The theory is inherently microbe-centric and does not account for variability in disease severity among individuals and asymptomatic carriership—two phenomena indicating an important role for host variability in infection outcome. The basic tenet of the germ theory was recently challenged, and a radically host-centric paradigm referred to as the “full-blown host theory” was proposed. According to this view, the pathogen is reduced to a passive environmental trigger, and the development of disease is instead due to pre-existing immunodeficiencies of the host. Here, we consider the factors that determine disease severity using established knowledge concerning evolutionary biology, microbial pathogenesis, and host–pathogen interactions. We note that the available data support a noncentric view that recognizes key roles for both the causative microbe and the host in dictating infection outcome.

The germ theory of disease was developed in the mid/late 19th century and is arguably the most important paradigm in the history of medicine. It states that microbial pathogens are responsible for causing infectious diseases, i.e., it denotes cause and effect. The health implications of the theory were revolutionizing, providing a scientific rationale for both preventing (vaccines and hygiene) and treating (antibiotics) communicable diseases. Koch’s postulates laid out an experimental approach to positively identify disease-causing agents and represent a cornerstone of the germ theory (1) (Box 1). However, the theory is microbe-centric by nature and therefore does not explain why different individuals infected by the same pathogen may experience very different disease severity or even no symptoms at all (asymptomatic carriership). This situation suggests that while the microbe is required to cause disease it is not sufficient to dictate infection outcome. It follows that variability in the status of the host must be considered, as pointed out by Dubos in 1955 when he expressed his “second thoughts on the germ theory” (2).

Box 1. Koch’s postulates as commonly described.

1. The microbe must be found in all individuals suffering from the disease, but not in healthy individuals.
2. The microbe must be isolated from a diseased individual and grown into a pure culture.
3. The cultured microbe should cause disease when introduced into a healthy experimental host.
4. The microbe must be reisolated from the experimental host and shown to be the same as the original.

Of note, Koch and other contemporary scientists soon realized the existence of asymptomatic carriership and excluded the requirement that the pathogen should not be found under healthy conditions.

In a recent reflection on infection outcome, Casanova challenged the germ theory and proposed a new host-centric paradigm, referred to as the “full-blown host theory,” in its place (3). According to this view, the microbe is equated to an environmental trigger that merely reveals underlying and preexisting immunodeficiencies of the host (3). Here, we examine the relative importance of pathogen and host in determining infection outcome and argue that it is imperative to adopt a noncentric view, acknowledging key roles for both the causative microbe and the host.

## The Role of Pathogen and Host in Infection Outcome

### The Full-Blown Host Theory.

The last decades have provided a wealth of information concerning the contribution of immunodeficiencies to infection outcome (4). It is in light of such insights that Casanova relegates the role of the microbe in infectious diseases to a passive environmental trigger akin to the role of a peanut in peanut allergy, and rhetorically asks “who would see peanut as the cause of peanut allergy?” (3). Casanova finds it bizarre that there are papers talking about “death from infection in an immunocompetent individual,” since it would be equivalent to talking about “death from respiratory failure in a patient with normal pulmonary function” or “death from coma in a patient with normal brain function” (3). Instead, the full-blown host theory states that infectious diseases are caused exclusively by inherited (“inborn errors of immunity”) or acquired immunodeficiencies of the host (3). These deficiencies can be “overt” or “covert” (i.e., undetectable) depending on the techniques available for their detection, and they “can only rigorously be defined by a severe infection” (3). As outlined below, we find the relevance of these comparisons and propositions to infection biology questionable, especially since infection involves two interacting organisms (5). We also note that the hypothesis regarding so-called covert immunodeficiencies—which are assumed to exist but remain unknown—represents an unscientific conjecture as it cannot be falsified.

### What Is a Pathogen?

To explore the role of pathogen and host in infection outcome, we first need to decide on what we consider a pathogen. A pathogen is usually defined as a microbe that is able to cause disease in an immunocompetent and otherwise healthy host. The concept of asymptomatic carriership, however, implies that pathogens can sometimes infect/colonize a host without producing symptomatic disease. Examples of this common phenomenon include SARS-CoV-2 and *Helicobacter pylori* where as much as ~40% and ~80%, respectively, of all infections may be asymptomatic (6, 7). The perhaps most significant example of a pathogen that can cause asymptomatic carriership is *Mycobacterium tuberculosis*, for which it is estimated that ~25% of the global human population is latently infected without showing any signs of disease (8), although the high prevalence of latency has recently been questioned (9). In contrast to pathogens, so-called opportunistic pathogens are considered unable to generate disease under normal conditions but are able to do so if the steady-state of the host is breached. For example, the spore-forming bacterium *Clostridium difficile*, which can be part of the gut microbiota, is normally unable to establish symptomatic infection but may do so upon treatment with antibiotics, which diminishes the microbiota thus enabling germinating *C. difficile* cells to gain a foothold (10). Commensal fungi of the *Candida* genus are similarly unable to cause disease under normal circumstances but can do so if the steady-state is breached by immunosuppression, as in patients with AIDS (11). Indeed, it is well established that both inherited and acquired immunodeficiencies can lead to opportunistic infections, as well as to worsened outcome of infections with a pathogen.

The discussion above suggests that the distinctions between pathogens, opportunistic pathogens, and benign members of the microbiota can be debated and that these terms might rather reflect a continuum. Still, pathogens are distinguished by their capacity to cause disease in healthy immunocompetent host individuals, a feature dependent on evolved virulence traits.

### The Evolution of Virulence.

Virulence denotes the ability of a pathogen to cause disease symptoms/pathology and is a key aspect of infection outcome. Up until the 1980s, it was widely believed that all pathogens would gradually loose virulence and evolve into commensals. However, based on evolutionary theory initially developed by Anderson and May (12), and subsequently extended by numerous authors (13), it is now thought that the Darwinian fitness of a pathogen is typically maximized at some intermediate level of virulence. This is because virulence entails both fitness costs in the form of, for example, host death leading to premature truncation of transmission, and benefits in the form of increased between-host transmission rate (12). The optimal level of virulence from the pathogen’s perspective is determined by factors like transmission mode, extent of within-host competition, etc (14). The theory is supported by comprehensive tests in both controlled laboratory experiments, including rodent malaria (15), and epidemiological studies of human disease, such as HIV/AIDS (16). Thus, natural selection has shaped pathogens to cause symptomatic disease in the general host population to enhance between-host transmission. Virulence can also be affected by short-sighted within-host evolution, where mutations providing an advantage in the tissue or allowing spread into a new replicative niche—distinct from the original site of infection—are selected for even though they confer no benefit to between-host transmission (17). From an evolutionary perspective, pathogens are therefore expected to express factors that promote the development of symptomatic disease in immunocompetent host individuals, either as a result of selection for enhanced between-host transmission or by short-sighted within-host adaptation.

### Virulence Factors and Host–Pathogen Interactions.

To illustrate how pathogens cause disease and to highlight the fundamental difference between the role of a pathogen in infection and, for example, the role of a peanut in allergy, we will focus on *Streptococcus pyogenes* as a model. Humans represent its only known replicative niche (18), meaning that the bacterium evolves exclusively within us. Infection with *S. pyogenes* can lead to asymptomatic carriership as well as to symptomatic diseases, ranging from superficial throat and skin infections to invasive and life-threatening conditions like necrotizing soft tissue infection and septic shock (18, 19). Globally, *S. pyogenes* is responsible for ~700 million cases of disease and over 500,000 deaths annually (18, 19). We have selected a few concrete examples demonstrating the evolved ability of this major human pathogen to actively promote disease in immunocompetent individuals.

**Fig. 1.**
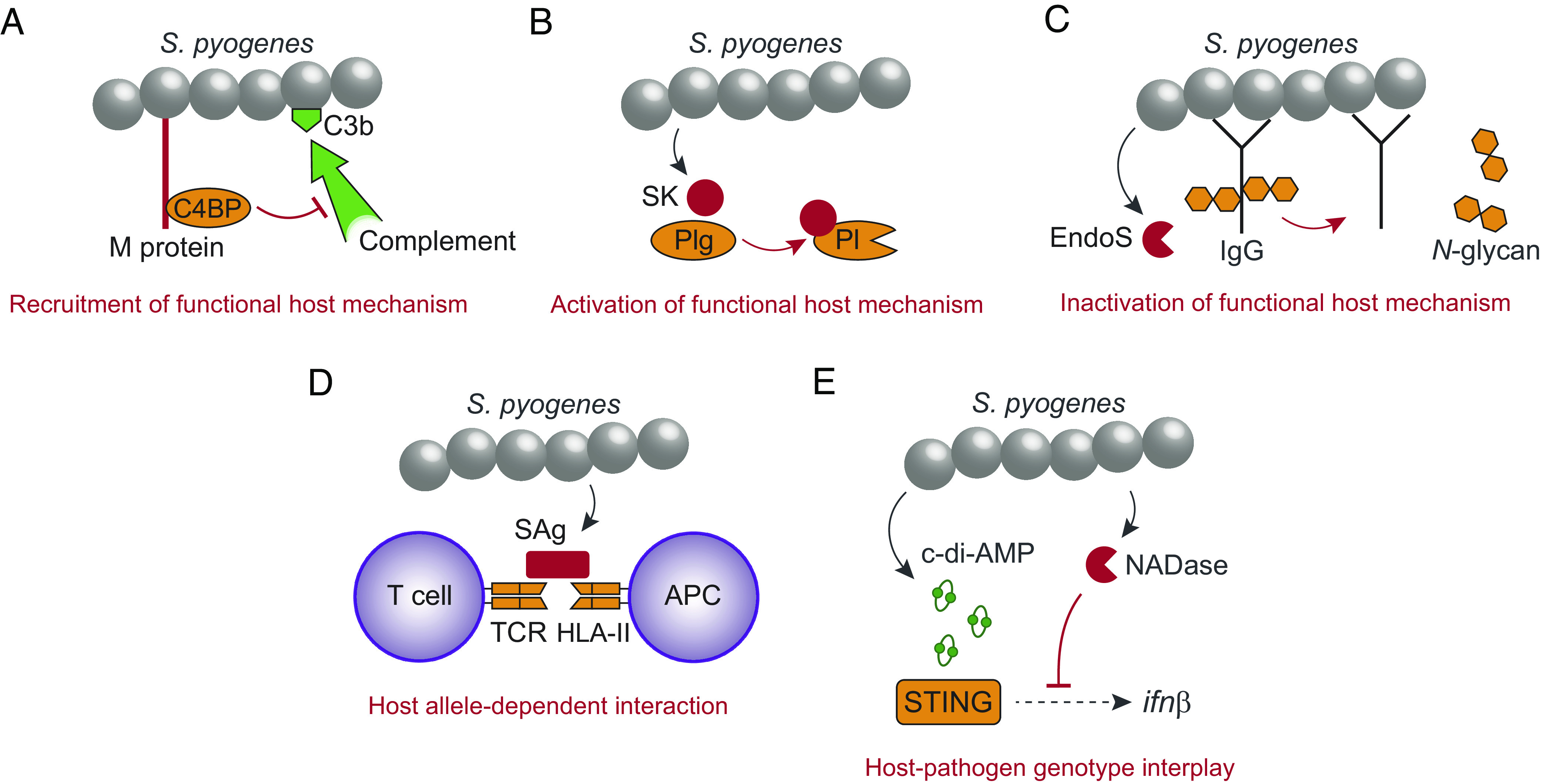
Examples demonstrating the evolved ability of *S. pyogenes* to promote disease in immunocompetent individuals. (*A*) The surface M protein recruits human C4BP to inhibit complement opsonization (C3b) of the bacterial surface. (*B*) Secreted streptokinase (SK) binds to human Plg, which causes a conformational change of Plg into a plasmin (Pl) active state. (*C*) The secreted endoglycosidase EndoS inactivates effector functions of IgG by cleaving off *N*-glycans from the Fc-region. (*D*) Secreted superantigen (SAg) causes antigen-independent T cell activation by cross-linking the TCR with HLA-II on antigen presenting cells (APC). SAgs have different affinity for different fully functional HLA-II haplotypes. (*E*) The STING responds to *S. pyogenes*-derived c-di-AMP to induce transcription of the interferon β gene, which is inhibited by the enzymatic activity of bacterial NADase. Human STING and *S. pyogenes* NADase exhibit polymorphisms affecting their relative ability to respond to c-di-AMP and to suppress interferon transcription, respectively.

The surface M protein of *S. pyogenes* is a polymorphic virulence factor that confers resistance to phagocytosis, at least in part by inhibiting opsonization by complement (20). To this end, the M protein of many clinical strains recruits the plasma protein C4b-binding protein (C4BP), which upon binding to M protein maintains its normal function to inhibit complement activation, thereby preventing opsonophagocytosis and enabling rapid bacterial growth in human blood (21–23) (Fig. 1*A*). These M proteins selectively bind C4BP of human origin and analysis of transgenic mice has confirmed a critical role for this host–pathogen interaction in virulence (24, 25). Thus, *S. pyogenes* exploits the normal function of a complement regulator to generate a complement-deficient microenvironment in a complement-proficient host.

Our second example involves streptokinase, a secreted *S. pyogenes* protein that selectively binds to and activates human plasminogen (Plg) to promote proteolysis and bacterial dissemination (18, 26) (Fig. 1*B*). Infection of transgenic mice has demonstrated a key role for this interaction in virulence (26), showing that *S. pyogenes* activates the normal function of the fibrinolytic system to promote disease. The fact that the bacterial interactions with C4BP and Plg are host-specific is of central importance, because it essentially excludes the possibility that these virulence traits have evolved for other purposes outside of the human host.

A third example is provided by the endoglycosidase EndoS, which is secreted from *S. pyogenes* and cleaves the conserved *N*-glycan on immunoglobulin G (IgG) antibodies (27) (Fig. 1*C*). Removal of this glycan from the Fc-region impairs IgG effector functions, including ability to activate complement and interact with Fc-receptors to induce phagocytosis (28). Lack of EndoS has no effect on virulence under naive conditions but significantly limits disease development in immunized mice (29). Thus, the bacterium has evolved a mechanism to inactivate the protective function of specific IgG antibodies in an immunocompetent host.

Our remaining two examples showcase the importance of an interplay between bacterial virulence factors and host genetic variability. First, streptococcal superantigens (SAgs) cross-link T cell receptors (TCRs) with human MHC class II [human leukocyte antigen II (HLA-II)] molecules leading to antigen-independent T cell activation, which may cause a cytokine storm and shock (18) (Fig. 1*D*). However, patients carrying the HLA-DRB1*1501/DQB1*0602 haplotype have reduced responses to SAgs, rendering them less likely to develop severe systemic inflammation compared to individuals carrying risk or neutral haplotypes (30). Thus, different fully functional HLA-II variants have significant impact on infection outcome. From an evolutionary point of view, SAgs long remained enigmatic since the ability to induce life-threatening systemic responses is unlikely to provide a selective advantage for the bacteria. Recent advances based on a noninvasive infection model in HLA-II transgenic mice, however, have suggested that streptococcal SAgs modulate the local immune response at mucosal surfaces to promote colonization of the nasopharynx (31, 32), offering a plausible explanation to how these virulence factors increase bacterial fitness. The fact that SAgs sometimes provoke systemic disease may therefore be viewed as an accidental consequence of their evolved ability to promote mucosal colonization. It is interesting to speculate that similar scenarios might apply more broadly to the role of virulence factors in disease development, particularly regarding pathogens associated with asymptomatic carriership.

The final example entails type I interferon (IFN)-signaling, which protects against host-detrimental inflammation in *S. pyogenes* infected mice (33, 34). In macrophages, *S. pyogenes*-derived cyclic-di-AMP (c-di-AMP) activates the stimulator of interferon genes (STING) to drive the type I IFN response (35, 36) (Fig. 1*E*). However, so-called epidemic strains of the bacterium secrete an enzymatically active NAD-glycohydrolase (NADase) (37–39) that suppresses type I IFN transcription (36) (Fig. 1*E*). Human STING and *S. pyogenes* NADase exhibit polymorphisms affecting their ability to respond to c-di-AMP (40–44) and suppress type I IFN production (36), respectively. Paired analyses of patients and patient-derived strains indicate that an interplay between STING genotype and NADase activity regulates the outcome of invasive necrotizing soft tissue infections (36), suggesting that the particular combination of host and bacterial allele variants in each individual infection is consequential.

The examples above demonstrate that microbial virulence factors promote disease in immunocompetent host individuals. While we have focused on *S. pyogenes*, similar strategies to recruit, activate and inactivate functional aspects of the host response are widespread among microbial pathogens, including evolutionarily diverse bacterial species (45, 46), parasites (47), and viruses (48).

### Host and Pathogen Genetic Variability.

The inborn errors of immunity invoked by Casanova as the main cause of severe disease are the result of rare mutations (allele frequency ≤1%) with large effects on infection outcome (49). Although such mutations clearly play a role—for example, inborn errors affecting type I IFN production explain 3.5% of all cases with life-threatening COVID-19 (50)—more common alleles with smaller effects also make substantial contributions to the phenotypic variation in infection outcome, as indicated by genome-wide association studies (51). In at least some cases, such alleles are maintained by balancing selection, where alternative alleles at a locus are subject to opposing selection pressures (52). These variants can therefore not be characterized as inborn errors or primary immunodeficiencies but rather represent adaptive genetic variation. The best-known examples include the sickle cell trait, which affects susceptibility to severe malaria (53, 54), and different HLA variants affecting susceptibility to severe disease from infection with numerous pathogens (51). Other examples of the effects of balancing selection on infection outcome include *ABO*, *CCR5*, *FUT2*, and *TIRAP* (51, 55–57).

Besides host genetic variation, there is often extensive genetic variation in pathogens, and genetic diversity of host and pathogen may combine in a nonadditive way such that the specific combination of host and pathogen genotypes significantly affects disease severity (52). Thus, while host genetics clearly plays a role, it is important not to limit this to inborn errors of immunity. We also need to recognize the effect of adaptive genetic variation in hosts and interactions between host and pathogen genetic diversity, the very basis for coevolution (58).

### René Dubos and the Role of Genetic Factors in Infection.

René Dubos (1901 to 1982) was one of the great pioneers of modern microbiology. In the 1955 article referred to above (2), Dubos focused on how changing circumstances—such as nutritional status or acquisition of (noninfectious) diseases—may affect the status of the host and infection outcome, but he did not consider the role of host genetics. Casanova criticizes Dubos for neglecting the importance of inheritance in this and in subsequent work and for not concluding that immunodeficiency is the main factor determining the outcome of an infection, as per the full-blown host theory (3). It is argued that Dubos “was not clairvoyant or bold enough to envisage or speak about such a revolution” (3). This criticism is surprising to us since it is evident from chapter VII in Dubos’ book “Man Adapting,” first published in 1965, that he was well aware of the role of host genetics in infection (59), as reported in seminal work by Allison (54) and Webster (60) during the 1930 to 1950s. Dubos also discussed the classical rabbit myxomatosis studies (61), which demonstrated adaptations of both pathogen and host, and stated that “it seems reasonable to believe, but difficult to prove, that genetic changes also occur in the resistance of man to his pathogens.” The arguments put forward by Dubos (2, 59) indicate that he acknowledged a role for host genetics and immunity but that he likely favored a noncentric view on infection biology.

## Conclusions

Throughout evolutionary history microbes have adapted to thrive in various harsh environments, including the arctic ice, hot vents on the seafloor, sulfur-acidic lakes, and immunocompetent host individuals. Our review of well-established research emphasizes that pathogens, unlike environmental triggers, have evolved specific strategies to harness and evade functional host responses, promoting their ability to establish infection and cause symptomatic disease in immunocompetent individuals. It also underlines that infection outcome can be a product of an interplay between genetic diversity in the host and in the pathogen, indicating that both organisms play active roles. As such, we effectively falsify the universality of the recently proposed full-blown host theory and, therefore, its value as an intellectual framework in which to understand infection biology. Instead, the available data support a noncentric view that recognizes key roles for both the causative microbe and the host in dictating infection outcome.

## Data Availability

There are no data underlying this work.

## References

[1] L. S. King, Dr. Koch’s postulates. J. Hist. Med. Allied Sci. **7**, 350–361 (1952). 12990783 10.1093/jhmas/vii.4.350

[2] R. J. Dubos, Second thoughts on the germ theory. Sci. Am. **193**, 31–35 (1955).

[3] J. L. Casanova, From second thoughts on the germ theory to a full-blown host theory. Proc. Natl. Acad. Sci. U.S.A. **120**, e2301186120 (2023). 37307437 10.1073/pnas.2301186120 PMC10293828

[4] J. L. Casanova, L. Abel, From rare disorders of immunity to common determinants of infection: Following the mechanistic thread. Cell **185**, 3086–3103 (2022). 35985287 10.1016/j.cell.2022.07.004 PMC9386946

[5] M. Sironi, R. Cagliani, D. Forni, M. Clerici, Evolutionary insights into host-pathogen interactions from mammalian sequence data. Nat. Rev. Genet. **16**, 224–236 (2015). 25783448 10.1038/nrg3905 PMC7096838

[6] G. Syangtan , Asymptomatic SARS-CoV-2 carriers: A systematic review and meta-analysis. Front. Public Health **8**, 587374 (2020). 33553089 10.3389/fpubh.2020.587374 PMC7855302

[7] P. Malfertheiner , *Helicobacter pylori* infection. Nat. Rev. Dis. Primers **9**, 19 (2023). 37081005 10.1038/s41572-023-00431-8 PMC11558793

[8] M. Pai , Tuberculosis. Nat. Rev. Dis. Primers **2**, 16076 (2016). 27784885 10.1038/nrdp.2016.76

[9] M. A. Behr, E. Kaufmann, J. Duffin, P. H. Edelstein, L. Ramakrishnan, Latent tuberculosis: Two centuries of confusion. Am. J. Respir. Crit. Care Med. **204**, 142–148 (2021). 33761302 10.1164/rccm.202011-4239PP PMC8650795

[10] W. K. Smits, D. Lyras, D. B. Lacy, M. H. Wilcox, E. J. Kuijper, *Clostridium difficile* infection. Nat. Rev. Dis. Primers **2**, 16020 (2016). 27158839 10.1038/nrdp.2016.20 PMC5453186

[11] M. G. Netea, L. A. Joosten, J. W. van der Meer, B. J. Kullberg, F. L. van de Veerdonk, Immune defence against *Candida* fungal infections. Nat. Rev. Immunol. **15**, 630–642 (2015). 26388329 10.1038/nri3897

[12] R. M. Anderson, R. M. May, Coevolution of hosts and parasites. Parasitology **85**, 411–426 (1982). 6755367 10.1017/s0031182000055360

[13] S. Alizon, A. Hurford, N. Mideo, M. Van Baalen, Virulence evolution and the trade-off hypothesis: History, current state of affairs and the future. J. Evol. Biol. **22**, 245–259 (2009). 19196383 10.1111/j.1420-9101.2008.01658.x

[14] S. A. Frank, Models of parasite virulence. Q. Rev. Biol. **71**, 37–78 (1996). 8919665 10.1086/419267

[15] M. J. Mackinnon, A. F. Read, Virulence in malaria: An evolutionary viewpoint. Philos. Trans. R. Soc. Lond. B Biol. Sci. **359**, 965–986 (2004). 15306410 10.1098/rstb.2003.1414 PMC1693375

[16] C. Fraser , Virulence and pathogenesis of HIV-1 infection: An evolutionary perspective. Science **343**, 1243727 (2014). 24653038 10.1126/science.1243727 PMC5034889

[17] B. R. Levin, J. J. Bull, Short-sighted evolution and the virulence of pathogenic microorganisms. Trends Microbiol. **2**, 76–81 (1994). 8156275 10.1016/0966-842x(94)90538-x

[18] S. Brouwer , Pathogenesis, epidemiology and control of group A streptococcus infection. Nat. Rev. Microbiol. **21**, 431–447 (2023). 36894668 10.1038/s41579-023-00865-7 PMC9998027

[19] J. R. Carapetis, A. C. Steer, E. K. Mulholland, M. Weber, The global burden of group A streptococcal diseases. Lancet Infect. Dis. **5**, 685–694 (2005). 16253886 10.1016/S1473-3099(05)70267-X

[20] V. A. Fischetti, Streptococcal M protein: Molecular design and biological behavior. Clin. Microbiol. Rev. **2**, 285–314 (1989). 2670192 10.1128/cmr.2.3.285 PMC358122

[21] K. Berggård , Binding of human C4BP to the hypervariable region of M protein: A molecular mechanism of phagocytosis resistance in *Streptococcus pyogenes*. Mol. Microbiol. **42**, 539–551 (2001). 11703674 10.1046/j.1365-2958.2001.02664.x

[22] F. Carlsson, K. Berggård, M. Stålhammar-Carlemalm, G. Lindahl, Evasion of phagocytosis through cooperation between two ligand-binding regions in *Streptococcus pyogenes* M protein. J. Exp. Med. **198**, 1057–1068 (2003). 14517274 10.1084/jem.20030543 PMC2194224

[23] J. Persson, B. Beall, S. Linse, G. Lindahl, Extreme sequence divergence but conserved ligand-binding specificity in *Streptococcus pyogenes* M protein. PLoS Pathog. **2**, e47 (2006). 16733543 10.1371/journal.ppat.0020047 PMC1464397

[24] D. Ermert , Virulence of group A streptococci is enhanced by human complement inhibitors. PLoS Pathog. **11**, e1005043 (2015). 26200783 10.1371/journal.ppat.1005043 PMC4511809

[25] J. Persson, G. Lindahl, Single-step purification of human C4b-binding protein (C4BP) by affinity chromatography on a peptide derived from a streptococcal surface protein. J. Immunol. Methods **297**, 83–95 (2005). 15777933 10.1016/j.jim.2004.11.024

[26] H. Sun , Plasminogen is a critical host pathogenicity factor for group A streptococcal infection. Science **305**, 1283–1286 (2004). 15333838 10.1126/science.1101245

[27] M. Collin, A. Olsen, EndoS, a novel secreted protein from *Streptococcus pyogenes* with endoglycosidase activity on human IgG. EMBO J. **20**, 3046–3055 (2001). 11406581 10.1093/emboj/20.12.3046 PMC150189

[28] M. Collin , EndoS and SpeB from *Streptococcus pyogenes* inhibit immunoglobulin-mediated opsonophagocytosis. Infect Immun. **70**, 6646–6651 (2002). 12438337 10.1128/IAI.70.12.6646-6651.2002 PMC133027

[29] A. Naegeli , *Streptococcus pyogenes* evades adaptive immunity through specific IgG glycan hydrolysis. J. Exp. Med. **216**, 1615–1629 (2019). 31092533 10.1084/jem.20190293 PMC6605743

[30] M. Kotb , An immunogenetic and molecular basis for differences in outcomes of invasive group A streptococcal infections. Nat. Med. **8**, 1398–1404 (2002). 12436116 10.1038/nm1202-800

[31] K. J. Kasper , Bacterial superantigens promote acute nasopharyngeal infection by *Streptococcus pyogenes* in a human MHC class II-dependent manner. PLoS Pathog. **10**, e1004155 (2014). 24875883 10.1371/journal.ppat.1004155 PMC4038607

[32] J. J. Zeppa , Nasopharyngeal infection by *Streptococcus pyogenes* requires superantigen-responsive Vbeta-specific T cells. Proc. Natl. Acad. Sci. U.S.A. **114**, 10226–10231 (2017). 28794279 10.1073/pnas.1700858114 PMC5617250

[33] N. Gratz , Type I interferon production induced by *Streptococcus pyogenes*-derived nucleic acids is required for host protection. PLoS Pathog. **7**, e1001345 (2011). 21625574 10.1371/journal.ppat.1001345 PMC3098218

[34] V. Castiglia , Type I interferon signaling prevents IL-1beta-driven lethal systemic hyperinflammation during invasive bacterial infection of soft tissue. Cell Host Microbe **19**, 375–387 (2016). 26962946 10.1016/j.chom.2016.02.003

[35] E. Movert , Streptococcal M protein promotes IL-10 production by cGAS-independent activation of the STING signaling pathway. PLoS Pathog. **14**, e1006969 (2018). 29579113 10.1371/journal.ppat.1006969 PMC5886698

[36] E. Movert , Interplay between human STING genotype and bacterial NADase activity regulates interindividual disease variability. Nat. Commun. **14**, 4008 (2023). 37414832 10.1038/s41467-023-39771-0 PMC10326033

[37] P. Sumby , Evolutionary origin and emergence of a highly successful clone of serotype M1 group A streptococcus involved multiple horizontal gene transfer events. J. Infect. Dis. **192**, 771–782 (2005). 16088826 10.1086/432514

[38] W. Nasser , Evolutionary pathway to increased virulence and epidemic group A streptococcus disease derived from 3,615 genome sequences. Proc. Natl. Acad. Sci. U.S.A. **111**, E1768–1776 (2014). 24733896 10.1073/pnas.1403138111 PMC4035937

[39] L. Zhu , A molecular trigger for intercontinental epidemics of group A streptococcus. J. Clin. Invest. **125**, 3545–3559 (2015). 26258415 10.1172/JCI82478 PMC4588293

[40] E. J. Diner , The innate immune DNA sensor cGAS produces a noncanonical cyclic dinucleotide that activates human STING. Cell Rep. **3**, 1355–1361 (2013). 23707065 10.1016/j.celrep.2013.05.009 PMC3706192

[41] L. Jin , Identification and characterization of a loss-of-function human MPYS variant. Genes Immun. **12**, 263–269 (2011). 21248775 10.1038/gene.2010.75 PMC3107388

[42] S. Patel, L. Jin, TMEM173 variants and potential importance to human biology and disease. Genes Immun. **20**, 82–89 (2019). 29728611 10.1038/s41435-018-0029-9 PMC6212339

[43] M. M. Walker , Selective loss of responsiveness to exogenous but not endogenous cyclic-dinucleotides in mice expressing STING-R231H. Front. Immunol. **11**, 238 (2020). 32153571 10.3389/fimmu.2020.00238 PMC7049784

[44] G. Yi , Single nucleotide polymorphisms of human STING can affect innate immune response to cyclic dinucleotides. PLoS One **8**, e77846 (2013). 24204993 10.1371/journal.pone.0077846 PMC3804601

[45] A. S. Santos, B. B. Finlay, Bringing down the host: Enteropathogenic and enterohaemorrhagic *Escherichia coli* effector-mediated subversion of host innate immune pathways. Cell Microbiol. **17**, 318–332 (2015). 25588886 10.1111/cmi.12412

[46] P. Chandra, S. J. Grigsby, J. A. Philips, Immune evasion and provocation by *Mycobacterium tuberculosis*. Nat. Rev. Microbiol. **20**, 750–766 (2022). 35879556 10.1038/s41579-022-00763-4 PMC9310001

[47] M. Chulanetra, W. Chaicumpa, Revisiting the mechanisms of immune evasion employed by human parasites. Front. Cell Infect. Microbiol. **11**, 702125 (2021). 34395313 10.3389/fcimb.2021.702125 PMC8358743

[48] S. Ahmadi, M. Bazargan, R. Elahi, A. Esmaeilzadeh, Immune evasion of severe acute respiratory syndrome coronavirus-2 (SARS-CoV-2); molecular approaches. Mol. Immunol. **156**, 10–19 (2023). 36857806 10.1016/j.molimm.2022.11.020 PMC9684099

[49] J. L. Casanova, L. Abel, Lethal infectious diseases as inborn errors of immunity: Toward a synthesis of the germ and genetic theories. Annu. Rev. Pathol. **16**, 23–50 (2021). 32289233 10.1146/annurev-pathol-031920-101429 PMC7923385

[50] Q. Zhang , Inborn errors of type I IFN immunity in patients with life-threatening COVID-19. Science **370**, eabd4570 (2020). 32972995 10.1126/science.abd4570 PMC7857407

[51] A. J. Kwok, A. Mentzer, J. C. Knight, Host genetics and infectious disease: New tools, insights and translational opportunities. Nat. Rev. Genet. **22**, 137–153 (2021). 33277640 10.1038/s41576-020-00297-6 PMC7716795

[52] L. Råberg, Human and pathogen genotype-by-genotype interactions in the light of coevolution theory. PLoS Genet. **19**, e1010685 (2023). 37023017 10.1371/journal.pgen.1010685 PMC10079023

[53] G. Band , Malaria protection due to sickle haemoglobin depends on parasite genotype. Nature **602**, 106–111 (2022). 34883497 10.1038/s41586-021-04288-3 PMC8810385

[54] A. C. Allison, Protection afforded by sickle-cell trait against subtertian malareal infection. Br. Med. J. **1**, 290–294 (1954). 13115700 10.1136/bmj.1.4857.290 PMC2093356

[55] M. J. Bamshad , A strong signature of balancing selection in the 5’ cis-regulatory region of CCR5. Proc. Natl. Acad. Sci. U.S.A. **99**, 10539–10544 (2002). 12149450 10.1073/pnas.162046399 PMC124967

[56] A. Ferrer-Admetlla , A natural history of FUT2 polymorphism in humans. Mol. Biol. Evol. **26**, 1993–2003 (2009). 19487333 10.1093/molbev/msp108

[57] C. C. Khor , A Mal functional variant is associated with protection against invasive pneumococcal disease, bacteremia, malaria and tuberculosis. Nat. Genet. **39**, 523–528 (2007). 17322885 10.1038/ng1976 PMC2660299

[58] D. Ebert, P. D. Fields, Host-parasite co-evolution and its genomic signature. Nat. Rev. Genet. **21**, 754–768 (2020). 32860017 10.1038/s41576-020-0269-1

[59] R. J. Dubos, Man Adapting (Yale University Press, 1980), p. 527.

[60] L. T. Webster, Experimental epidemiology. Medicine (Baltimore) **25**, 77–109 (1946). 21014710 10.1097/00005792-194602000-00003

[61] F. Fenner, “Myxomatosis in Australian wild rabbits–Evolutionary changes in an infectious disease” in The Harvey Lectures (Academic press, New York, 1959), pp. 25–55. 13640418

